# Toward an Integrated Model of Health Sciences Academies in Nepal: Policy Rationale, Benefits, and Challenges

**DOI:** 10.31729/jnma.9195

**Published:** 2025-08-31

**Authors:** Shrikrishna Giri

**Affiliations:** 1 Tokha municipality, South Western College Marg, Basundhara, Kathmandu, Nepal

**Keywords:** *health sciences academies*, *medical education*, *medical education commission*, *quality health services*, *umbrella act*

## Abstract

Academies of Health Sciences have increased significantly in Nepal in the past decade to improve access to medical education and quality health services for the general public. They are established with their act. The provincial-level government also established health science academies in the past decade. They are mainly in the urban and geographically accessible regions of the country. There is a great demand for health sciences academies in areas where they do not exist. The Government of Nepal is going to establish the health science academies/ institutions according to the national priorities. This article analyzes the different views of the Integrated Health Sciences Act, commonly known as the Health Sciences Umbrella Act.

## INTRODUCTION

The Integrated Health Sciences Act, commonly known as the Health Sciences Umbrella Act, is a legislative framework aimed at unifying various health science academies and institutions under a single legal structure.^1^ To improve access to medical education and quality health services for the general public, the number of health science academies increased under the federal government and is rapidly growing in the provinces. Some of the province-level health science academies have already started the academic programs.^2^ The concept of decentralization of medical education and health services is being implemented. But the institutions are mainly established in the urban, highly populated, and geographically accessible regions. More than half a dozen institutions are running undergraduate and postgraduate health sciences programs.^2^ The Act of Parliament establishes them. And they are of different levels of physical, academic, and health service capabilities. The present scenario has deployed fragmented governance, inconsistent quality education, duplication of administration, inadequate use of physical infrastructure, underutilization of human resources, lengthy legal procedures to expand the health sciences institutions to the needy regions, and increased financial burden to the government. To address these challenges, the Ministry of Health and Population (MoHP), supported by the Medical Education Commission (MEC), has proposed the Integrated Health Sciences Act. The MEC had submitted the concept paper to the Nepal government upon the directive of the then-prime minister. The author reflects on the institutional reforms undertaken, and the rationale for the integrated academic system aligns with national priorities in this article.

## POLICY RATIONALE

National Medical Education Act (NMEA) was formulated to address the then-existing irregularities in fee structure, admission, eligibility to persue medical education, and access to the medical education by marginalized and low economic status students, disparity in the establishment of health science education institutions and produce human resources according to the national priorities there by making quality health services accessible to the general public. The Medical Education Commission is formed as per the National Medical Education Act (NMEA).^2^ It successfully addresses the issue of fee structure, admission based on merit, and eligibility to study within the country and abroad. Developed a national curriculum framework for undergraduate programs in medical education. These are examples of the integrated nature of activities undertaken by the MEC. With these examples functioning nationally, further harmonization among the academies becomes logical.

Health science academies established to produce a high-level health workforce according to the national priorities are functioning under their act. More than half a dozen academies are conducting undergraduate and postgraduate programs.^2^ They are of different levels of capacities in physical structure, academic activities, health service delivery, and catering health services to the district and community levels. They have separate administrative, educational, and financial structures and autonomy. These academies are mainly situated in the urban, highly populated, and geographically accessible regions of the country. There are upcoming institutions, according to the national priorities. They are facing challenges to overcome not only the legal procedure but also the financial burden of the nation. To address these issues, the Ministry of Health and Population has proposed the Integrated Health Sciences Academies Act, which is commonly known as the umbrella act of health sciences academies.^1^ The rationale of the proposed act is to eliminate the duplication of administrative and academic functions, share the physical and educational resources, and improve the quality of education and health services. The Integrated Health Sciences Act will facilitate the establishment of the upcoming health science institution.

## BENEFITS

Integrated health sciences academies, a unified academic framework, ensure comparable graduate competencies, facilitating the recognition of international education standards. It increases the administrative efficiency and reduces costs by consolidating the governance system. It shares the faculty, Hospitals, and research facilities across provinces.

## CHALLENGES

Despite its advantages, it faces several challenges; There may be institutional resistance to perceiving the established academies as a threat to autonomy, transferring faculties, sharing resources, and compromising the existing facilities.^5^ The provincial authority may feel threatened in balancing provincial rights in the federal dynamics.

## DISCUSSION

The necessity of the National Medical Education Act was felt when the nation realized the quality of medical education was falling. The cost of education was skyrocketing. There was no admission system based on merit. There was no standard gate for the scholarship provided by a foreign country. There were no inclusive rules and regulations. The health sciences institutions were concentrated in the urban and densely populated region of the country. The rate of increase of the public institutions was very low. The Medical Education Commission streamlined most of the issues and laid the foundation for quality medical education in the country and abroad, implemented access to medical education, and distributed health science education to remote, needy regions of the country.^2^ Now is the time to integrate the health sciences academies under a unified national-level framework.^1^ The national level integrated health sciences institution will deliver quality medical education, reduce administrative costs, and share academic and physical infrastructure and human resources.^4^ It will also enable the establishment of institutions according to national priorities.^3^

We can learn good practices from international practices, too. All India Institute of Medical Sciences (AIIMS),^6^ New Delhi, oversees the institutions in different states of India. AIIMS demonstrates an example of how a centrally coordinated institution can maintain academic standards across diverse regions. UGC Sri Lanka governs all universities.^7^ Pakistan Medical Commission’s efforts are going on to streamline and improve accountability under a centralized regulatory mechanism.^8^

## CONCLUSIONS

Nepal’s medical education landscape is at a transformative juncture. The experience of MEC, particularly its success with the national common entrance exam and unified academic oversight, demonstrates the viability of integration. The proposed Umbrella Act provides a strategic pathway toward improving quality, reducing costs, and ensuring equitable access to health sciences education.^3^ With political commitment, legal clarity, and cooperative federalism, the integration of Nepal’s health sciences academies can become a model for sustainable academic governance in federal contexts.

## RECOMMENDATIONS

Due to the diversity in the establishment, the physical and academic capacity of the institution, the integration should be phased, beginning with functional areas such as curriculum harmonization, a common faculty development program, and joint academic councils. The stakeholders, representatives from provincial academies, teaching hospitals, faculty associations, and the students’ body, should be engaged in the transition process. The act should be finalized with explicit provisions for institutional autonomy, a transitional mechanism, and dispute resolution.
